# Positive SARS-CoV-2 detection on intraoperative nasopharyngeal viral testing is not associated with worse outcomes for asymptomatic elective surgical patients

**DOI:** 10.3389/fmed.2022.1065625

**Published:** 2022-12-21

**Authors:** Paul W. Clancy, Ziyad O. Knio, Zhiyi Zuo

**Affiliations:** ^1^Department of Anesthesiology, University of Virginia, Charlottesville, VA, United States; ^2^School of Medicine, University of Virginia, Charlottesville, VA, United States

**Keywords:** COVID-19, SARS-CoV-2, intraoperative, surgery, length of stay

## Abstract

**Background:**

It has been demonstrated that surgical patients with COVID-19 are at increased risk for postoperative complications. However, this association has not been tested in asymptomatic elective surgical patients.

**Methods:**

A retrospective cohort study among elective gynecological and spine surgery patients at a single tertiary medical center from July 2020 through April 2022 (*n* = 1,130) was performed. The primary endpoint was prolonged (>75th percentile for the corresponding surgical service) length of stay. Secondary endpoints included postoperative respiratory complications, duration of supplemental oxygen therapy, and other major adverse events. The association between SARS-CoV-2 detection and the above outcomes was investigated with univariate and multivariable analyses.

**Findings:**

Of 1,130 patients who met inclusion criteria, 30 (2.7%) experienced intraoperative detection of SARS-CoV-2. Those with intraoperative viral detection did not experience an increased incidence of prolonged length of stay [16.7% vs. 23.2%; RR, 0.72 (95% CI, 0.32–1.61); *P* = 0.531] nor did they have a longer mean length of stay (4.1 vs. 3.9 days; *P* = 0.441). Rates of respiratory complications [3.3% vs. 2.9%; RR, 1.15 (95% CI, 0.16–8.11); *P* = 0.594] and mean duration of supplemental oxygen therapy (9.7 vs. 9.3 h; *P* = 0.552) were similar as well. All other outcomes were similar in those with and without intraoperative detection of SARS-CoV-2 (all *P* > 0.05).

**Interpretation:**

Asymptomatic patients with incidental detection of SARS-CoV-2 on intraoperative testing do not experience disproportionately worse outcomes in the elective spine and gynecologic surgical population.

## 1 Introduction

The COVID-19 pandemic has had significant effects on health systems worldwide (1). Millions of elective surgeries were canceled or delayed during the initial peak disruption, and elective surgery case volumes were projected to decline even further for numerous reasons (2, 3). Patient safety is a noteworthy concern. Early studies had demonstrated that surgical patients with SARS-CoV-2 experienced high rates of mortality and cardiopulmonary complications, perhaps higher than non-surgical patients hospitalized with SARS-CoV-2 (4–7). However, the relative risk of such complications in SARS-CoV-2 patients relative to healthy controls was not well-defined.

It is now understood that compared to patients without SARS-CoV-2, patients with SARS-CoV-2 are at increased risk of postoperative adverse events such as venous thromboembolism (8), pulmonary complications (9), and mortality (10). However, selection bias may adversely influence our current understanding. Postoperative complications in patients with diagnosed SARS-CoV-2 may be underestimated if elective surgery is only offered to patients whose respiratory symptoms are mild or absent. Conversely, if day-of-surgery viral testing is not mandated in asymptomatic individuals, then high-risk patients during the early inoculation stages of SARS-CoV-2 may remain undiagnosed. Regardless, intraoperative testing for SARS-CoV-2 is standard of practice at some institutions, including that of the authors. Although routine testing is relatively non-invasive and may yield epidemiologic benefits in limiting viral transmission both in the inpatient and post-discharge settings, the perioperative outcome improvements for surgical patients are yet to be quantified by substantial evidence.

This study aimed to investigate whether prolonged length of stay and other adverse outcomes were associated with intraoperative detection of SARS-CoV-2 in elective spine and gynecologic surgical patients. Notably, patients in this study were asymptomatic and provided documentation of a negative SARS-CoV-2 PCR test result within seven calendar days preoperatively. Intraoperative nasopharyngeal samples were collected from all patients immediately after induction of general anesthesia. The authors hypothesized that viral detection would be independently predictive of prolonged length of stay.

## 2 Materials and methods

### 2.1 Patient population, ethics approval

This retrospective cohort study investigated all elective spine and gynecologic surgeries performed at a single academic medical center from July 2020 through April 2022. The spine and gynecologic surgery patient population was selected due to its substantial elective case volume. Adjustments were made for the respective surgical services by defining service-specific endpoints and by controlling for surgical service during multivariable modeling, described in more detail in the methods to follow. All data were retrieved from the institutional electronic medical record, with queried variables collected *a priori* as part of routine care.

The study protocol (IRB-HSR# 23998) was approved by the Institutional Review Board with waiver of written consent (approval date: July 11, 2022).

### 2.2 Inclusion and exclusion criteria

After the initial data query, the following criteria were applied. Patients presenting from home for elective surgery with planned inpatient admission were included. Patients with preoperative laboratory confirmation of SARS-CoV-2 were excluded. During the study period, all elective surgical patients were required to provide documentation of a negative SARS-CoV-2 PCR test result within seven calendar days prior to arrival to the hospital on day of surgery. Patients were encouraged to have testing performed at the medical center, however, offsite testing was accepted if the testing was PCR (vs. antigen) detection per official report. Cases that were done under regional anesthesia (spinal, epidural, and/or peripheral nerve blockade), in contrast to general anesthesia, were excluded in order to maintain a more homogenous sample.

### 2.3 Measurements and data handling

The independent variable of interest was PCR detection of SARS-CoV-2 on intraoperative nasopharyngeal swab. At the authors’ institution, this is collected routinely for all patients presenting from home for elective surgery with planned inpatient admission. Anesthesiology case staff are trained in viral swab collection in a standardized fashion. The nasopharyngeal sample was harvested immediately after the induction of general anesthesia, and swabs were transported in a viral transport media for immediate on-site PCR testing (University of Virginia Hospital, institutional laboratory code: LAB4685). Specimens were stable for 24 h at room temperature and for 7 days if refrigerated. Results for surgical patients are typically made available within 2 h of collection.

Independent variables also included age, sex, body mass index (BMI), chronic obstructive pulmonary disease (COPD) diagnosis, diabetes diagnosis, hypertension diagnosis, preoperative serum hemoglobin concentration, preoperative serum creatinine concentration, American Society of Anesthesiologists (ASA) physical status classification, operative duration, and estimated blood loss. Surgical service (spine vs. gynecologic) was considered during the multivariable procedure.

### 2.4 Data statement

Data cannot be made publicly available for legal or ethical reasons.

### 2.5 Primary and secondary endpoints

The primary endpoint was hospital length of stay. A service-specific prolonged length of stay was defined as an inpatient surgical admission exceeding the 75th percentile of hospital admissions for the primary surgical service (spine vs. gynecologic), a discretization scheme that is consistent with what has been done previously (11).

Secondary endpoints included postoperative respiratory complications, duration of supplemental oxygen therapy, sepsis, deep venous thrombosis, pulmonary embolism, myocardial infarction (within 30 days), stroke (within 30 days), unanticipated intensive care unit (ICU) admission, reintubation, and mortality.

Some endpoints, including hospital length of stay, duration of supplemental oxygen therapy, ICU admission, reintubation, and mortality, were discrete elements captured in the electronic medical record.

Respiratory complications, sepsis, deep venous thrombosis, pulmonary embolism, myocardial infarction, and stroke were queried by standardized ICD-10 codes. COPD, diabetes, and hypertension diagnoses were verified similarly (Supplementary Table 1).

### 2.6 Sample size calculation

An *a priori* power analysis was conducted (12). Given that 25% of patients were expected to have a prolonged length of stay under the discretization scheme by quartile, the authors sought for the study to be adequately powered to detect a difference between 20% of control patients vs. 30% of SARS-CoV-2 positive patients experiencing prolonged length of stay. This corresponded to an effect size of *h* = 0.232; detecting this difference with 80% power required a sample size of *n* > 292. The final sample size obtained by applying the above inclusion and exclusion criteria to this observational study of consecutive cases acceptably exceeded this minimum value. Of note, the prevalence of SARS-CoV-2 intraoperative detection was unknown.

### 2.7 Statistical analyses

Statistical analysis was performed with R version 4.2.0 (R Core Team, Vienna, Austria) (13). Continuous variables were summarized by mean (standard deviation), while categorical variables were summarized by frequency (%). All hypothesis tests were two-sided, with significance defined by α = 0.05.

Baseline differences between the cohorts with and without SARS-CoV-2 detection were investigated with univariate analyses. The Mann-Whitney *U*-Test was applied to continuous variables while Fisher’s Exact Test was applied to categorical variables, given that the anticipated event rate for endpoints would be rare. Associations between SARS-CoV-2 detection and the primary and secondary endpoints were also investigated with univariate analyses. Relative risks (RR) and mean differences (MD) with 95% confidence intervals (CI) are reported. Survival differences were additionally explored with Kaplan-Meier analysis comparing the time to mortality against the detection of SARS-CoV-2 (14, 15).

A multivariable analysis was then conducted, modeling prolonged hospital length of stay against SARS-CoV-2 detection and/or other relevant predictors. Mixed effects modeling was employed to adjust for the effect of the primary surgical service (spine vs. gynecologic) as a random effects term. All other preoperative and intraoperative variables demonstrating marginal association (*P* < 0.10) with hospital length of stay on univariate analysis were treated as fixed effects. Variable selection was accomplished by backward stepwise model adjustment by Akaike information criterion. Model discrimination was assessed with a c-statistic, and independent predictors were summarized by an adjusted odds ratio (AOR) and accompanying 95% CI (16). The multivariable procedure was repeated on any secondary endpoints demonstrating an event rate of *n* > 30.

## 3 Results

### 3.1 Baseline characteristics

The inclusion and exclusion criteria yielded a sample of 1,130 patients. Of these, 30 patients (2.7%) had intraoperative detection of SARS-CoV-2. Compared to those without detection of SARS-CoV-2, those with intraoperative detection of SARS-CoV-2 were younger [47.3 vs. 58.4 years; MD, −11.08 (95% CI, −18.00 to −3.00); *P* = 0.006] but did not differ in any other preoperative or operative characteristics.

### 3.2 Primary endpoint

A prolonged length of stay corresponded to an inpatient gynecologic surgical admission greater than 3 days, or an inpatient spine surgical admission greater than 5 days.

### 3.3 Univariate analyses of all endpoints

Those with intraoperative detection of SARS-CoV-2 did not experience increased incidence of prolonged length of stay [16.7% vs. 23.2%; RR, 0.72 (95% CI, 0.32–1.61); *P* = 0.531] nor did they have a longer mean length of stay [4.1 vs. 3.9 days; *MD*, 0.15 (95% CI, 0.00–1.00); *P* = 0.441]. Respiratory complications were the only additional endpoint observed to have an event rate of *n* > 30. However, those with intraoperative detection did not experience increased incidence of respiratory complications [3.3% vs. 2.9%; RR 1.15 (95% CI, 0.16–8.11); *P* = 0.594], nor did they have a longer duration of supplemental O_2_ therapy [9.7 vs. 9.3 h; *MD*, 0.44 (95% CI, −0.57 to 0.26); *P* = 0.552]. All other endpoint incidences were similar in those with and without intraoperative detection of SARS-CoV-2 (all *P* > 0.05) (Table 1).

**TABLE 1:** Demographic data and univariate analyses of SARS-CoV-2 detection.

	No./total (%)				
Characteristic	Aggregate (*n* = 1,130)	None detected (*n* = 1,100)	Detected (*n* = 30)	*P*-value	Mean difference (MD) or relative risk (RR) (95% CI)
**Preoperative**					
Age, mean (SD), years	58.1 (16.2)	58.4 (16.0)	47.3 (21.2)	0.006	MD −11.08 (−18.00 to −3.00)
Female sex	668/1,129 (59.2)	650/1,100 (59.1)	18/29 (62.1)	0.849	RR 1.05 (0.79–1.40)
BMI, mean (SD), kg/m^2^	30.4 (7.3)	30.4 (7.3)	30.1 (8.1)	0.659	MD −0.39 (−3.21 to 2.11)
COPD	75/1,130 (6.6)	73/1,100 (6.6)	2/30 (6.7)	>0.999	RR 1.00 (0.26–3.90)
Diabetes	233/1,130 (20.6)	228/1,100 (20.7)	5/30 (16.7)	0.819	RR 0.80 (0.36–1.80)
Hypertension	511/1,130 (45.2)	498/1,100 (45.3)	13/30 (43.3)	0.855	RR 0.96 (0.63–1.45)
Preoperative hemoglobin, mean (SD), g/dL	13.5 (1.9)	13.5 (1.9)	13.5 (1.9)	0.854	MD 0.05 (−0.70 to 0.70)
Preoperative creatinine, mean (SD), mg/dL	0.9 (0.5)	0.9 (0.5)	0.8 (0.2)	0.226	MD −0.10 (−0.10 to 0.00)
**Operative**					
ASA 3 or 4	556/1,130 (49.2)	543/1,100 (49.4)	13/30 (43.3)	0.581	RR 0.88 (0.58–1.33)
Case duration, mean (SD), h	3.7 (2.5)	3.6 (2.5)	4.4 (2.3)	0.056	MD 0.78 (−0.02 to 1.43)
EBL, mean (SD), L	0.5 (0.6)	0.5 (0.6)	0.5 (0.6)	0.835	MD 0.02 (−0.10 to 0.15)
Spine service	912/1,130 (80.7)	889/1,100 (80.8)	23/30 (76.7)	0.638	RR 0.95 (0.78–1.16)
**Outcomes**					
Prolonged LOS	260/1,130 (23.0)	255/1,100 (23.2)	5/30 (16.7)	0.513	RR 0.72 (0.32–1.61)
LOS, mean (SD), days	3.9 (3.2)	3.9 (3.2)	4.1 (2.6)	0.441	MD 0.15 (0.00–1.00)
Respiratory complications	33/1,130 (2.9)	32/1,100 (2.9)	1/30 (3.3)	0.594	RR 1.15 (0.16–8.11)
Duration of supplemental O_2_, mean (SD), h	9.3 (19.6)	9.3 (19.7)	9.7 (17.5)	0.552	MD 0.44 (−0.57 to 0.26)
Sepsis	5/1,130 (0.4)	5/1,100 (0.5)	0/30 (0.0)	>0.999	RR 0.00 (0.00 to NaN)
Pulmonary embolism	6/1,130 (0.5)	6/1,100 (0.5)	0/30 (0.0)	>0.999	RR 0.00 (0.00 to NaN)
Myocardial infarction	5/1,130 (0.4)	5/1,100 (0.5)	0/30 (0.0)	>0.999	RR 0.00 (0.00 to NaN)
Stroke	4/1,130 (0.4)	4/1,100 (0.4)	0/30 (0.0)	>0.999	RR 0.00 (0.00 to NaN)
Mortality	23/1,130 (2.0)	22/1,100 (2.0)	1/30 (3.3)	0.465	RR 1.67 (0.23–11.96)

ASA, american society of anesthesiologists; BMI, body mass index; CI, confidence interval; COPD, chronic obstructive pulmonary disease; EBL, estimated blood loss; LOS, length of stay.

ICU admission was unreliably captured by the study design, with only three patients (0.3%) documented as unanticipated ICU admits. All cases occurred in patients without detection of SARS-CoV-2. As such, this variable was subsequently excluded from analysis. Reintubation was also not captured by the methodologic parameters. The only documented cases of venous thrombosis occurred in patients who also experienced pulmonary embolism. The thrombosed veins were the right femoral vein and the left popliteal vein. As such, deep venous thrombosis was not examined as a separate endpoint.

### 3.4 Survival analysis

**FIGURE 1:**
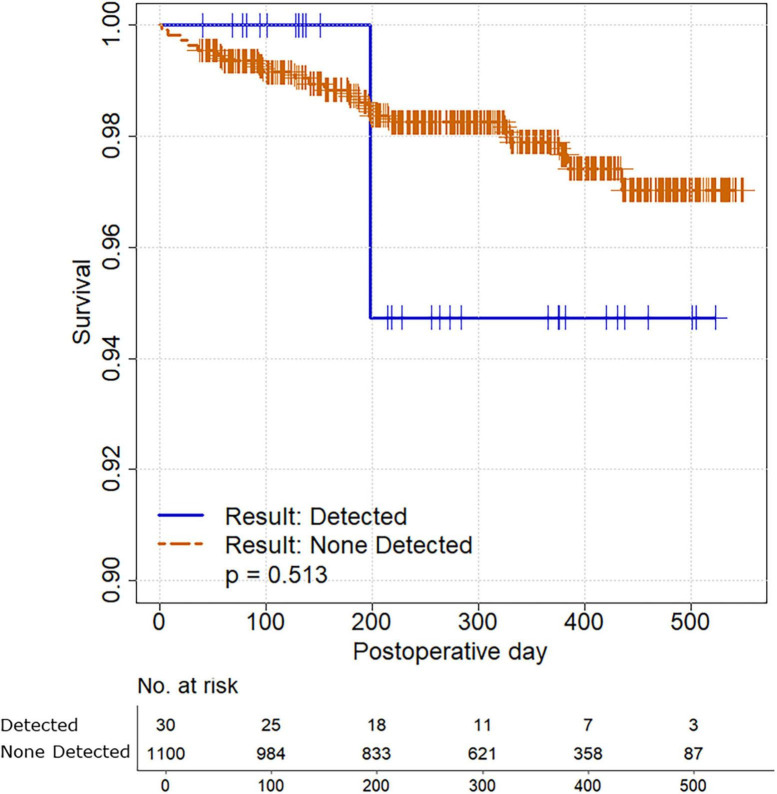
Survival analysis comparing study participants with and without intraoperative detection of SARS-CoV-2.

A total of 22 patients (2.0%) died during a median follow-up of 326 days (interquartile range 201–428). Only one decedent was in the cohort with intraoperative detection of SARS-CoV-2; survival time was not significantly different between the two cohorts (*P* = 0.513) (Figure 1).

### 3.5 Multivariate analyses of all endpoints

Potential predictors of prolonged length of stay and respiratory complications were explored. As stated in the methods, *P* < 0.10 was the criterion applied to identify potential fixed effects prior to stepwise model selection. Prolonged length of stay demonstrated a significant univariate association (*P* < 0.05) with age [*MD*, 4.59 years (95% CI, 2.31–6.87)], diabetes [RR, 1.36 (95% CI, 1.08–1.73)], preoperative hemoglobin [*MD*, −0.44 g/dL (95% CI, −0.71 to −0.17)], ASA class 3 or 4 [RR, 1.57 (95% CI, 1.26–1.96)], operative duration [*MD*, 1.15 h (95% CI, 0.83–1.46)], and estimated blood loss [*MD*, 0.51 L (95% CI, 0.37–0.65)]. Respiratory complications were significantly associated (*P* < 0.05) with diabetes [RR, 2.20 (95% CI, 1.10–4.41)] and marginally associated (*P* < 0.10) with ASA class 3 or 4 [RR, 1.81 (95% CI, 0.90–3.64)], operative duration [*MD*, 0.83 h (95% CI, −0.01 to 1.67)] and estimated blood loss [MD, 0.41 L (95% CI, 0.00–0.83)] (Table 2).

**TABLE 2:** Univariate analyses identifying potential predictors of prolonged length of stay and respiratory complications.

	Prolonged length of stay			Respiratory complications		
	No./total (%)			No./total (%)		
Characteristic	No (*n* = 870)	Yes (*n* = 260)	*P*-value	No (*n* = 1,097)	Yes (*n* = 33)	*P*-value
Age, mean (SD), years	57.0 (16.0)	61.6 (16.5)	<0.001	58.0 (16.2)	59.4 (18.2)	0.677
Female sex	509/870 (58.5)	159/259 (61.4)	0.407	644/1,096 (58.8)	24/33 (72.7)	0.108
BMI, mean (SD), kg/m^2^	30.3 (7.2)	30.8 (7.9)	0.433	30.4 (7.3)	31.5 (7.5)	0.417
COPD	59/870 (6.8)	16/260 (6.2)	0.721	72/1,097 (6.6)	3/33 (9.1)	0.565
Diabetes	165/870 (19.0)	68/260 (26.2)	0.012	221/1,097 (20.1)	12/33 (36.4)	0.023
Hypertension	388/870 (44.6)	123/260 (47.3)	0.441	498/1,097 (45.4)	13/33 (39.4)	0.495
Preoperative hemoglobin, mean (SD), g/dL	13.6 (1.9)	13.1 (1.9)	0.001	13.5 (1.9)	13.4 (2.2)	0.742
Preoperative creatinine, mean (SD), mg/dL	0.9 (0.4)	0.9 (0.6)	0.164	0.9 (0.5)	0.9 (0.3)	0.503
ASA 3 or 4	399/870 (45.9)	157/260 (60.4)	<0.001	535/1,097 (48.8)	21/33 (63.6)	0.092
Case duration, mean (SD), h	3.4 (2.5)	4.5 (2.2)	<0.001	3.6 (2.5)	4.5 (2.3)	0.053
EBL, mean (SD), L	0.4 (0.4)	0.9 (1.0)	<0.001	0.5 (0.6)	0.9 (1.1)	0.051
Surgical service: spine	700/870 (80.5)	212/260 (81.5)	0.699	883/1,097 (80.5)	29/33 (87.9)	0.289
SARS-CoV-2 result	25/870 (2.9)	5/260 (1.9)	0.403	29/1,097 (2.6)	1/33 (3.0)	0.892

ASA, American Society of Anesthesiologists; BMI, body mass index; COPD, chronic obstructive pulmonary disease; EBL, estimated blood loss.

Independent predictors of prolonged length of stay were age [AOR, 1.02 per year (95% CI, 1.00–1.03)], preoperative hemoglobin [AOR, 0.83 per g/dL (95% CI, 0.75–0.91)], ASA class 3 or 4 [AOR, 1.71 (95% CI, 1.18–2.49)], operative duration [AOR, 1.10 per hour (95% CI, 1.02–1.21)], and estimated blood loss [AOR, 3.11 per L (95% CI, 2.16–4.60)]. The prolonged length of stay predictive model demonstrated good discrimination (model c-statistic 0.743). Independent predictors of respiratory complications were diabetes [AOR, 2.46 (95% CI, 1.09–5.36)] and estimated blood loss [AOR, 1.80 per L (95% CI, 1.21–2.55)]. The respiratory complications predictive model demonstrated fair discrimination (model c-statistic 0.660) (Table 3).

**TABLE 3:** Multivariable analysis summary statistics for prolonged length of stay and respiratory complications.

	β (Standard error)	*P*-value	Adjusted odds ratio (95% CI)
**Prolonged length of stay predictors, model c = 0.743**			
Intercept	−0.87 (0.78)	0.265	0.42 (0.09–1.91)
Age, years	0.02 (0.01)	0.014	1.02 (1.00–1.03)
Preoperative hemoglobin, g/dL	−0.19 (0.05)	<0.001	0.83 (0.75–0.91)
ASA 3 or 4	0.54 (0.19)	0.005	1.71 (1.18–2.49)
Case duration, h	0.09 (0.04)	0.039	1.10 (1.02–1.21)
Estimated blood loss, L	1.14 (0.19)	<0.001	3.11 (2.16–4.60)
**Respiratory complications predictors, model c = 0.660**			
Intercept	−3.99 (0.30)	<0.001	0.02 (0.01–0.03)
Diabetes	0.90 (0.40)	0.025	2.46 (1.09–5.36)
Estimated blood loss, L	0.59 (0.19)	0.001	1.80 (1.21–2.55)

ASA, American Society of Anesthesiologists; CI, confidence interval.

## 4 Discussion

The present study demonstrates that asymptomatic elective surgical patients with positive intraoperative SARS-CoV-2 detection do not experience worse outcomes compared to controls. Specifically, the incidence of prolonged length of stay is not increased in patients with positive viral detection [RR, 0.72 (95% CI, 0.32–1.61)]. Although SARS-CoV-2 detection intraoperatively or immediately postoperatively may be too late to inform pre-operative and perioperative management strategies by the anesthesiology team and/or surgical team, our finding may have clinical relevance as institutions continually reassess the practice guidelines that inform whether intraoperative testing is clinically indicated. Similarly, this finding may provide reassurance to surgical centers that do not have the resources to perform routine testing on elective surgical patients.

Regardless, these findings may contribute to the growing body of evidence that can assist in determining SARS-CoV-2 surgical guidelines. For example, while the ASA initially recommended delaying of elective surgery for 4 weeks in order to minimize postoperative complications and mortality, Lieberman et al. (17) has since concluded that a 10-day delay of elective surgery from the time of symptoms or a positive SARS-CoV-2 test may be sufficient (17). However, recommendations such as these are primarily based upon minimizing viral transmission risks rather than mitigating an inherently high-risk postoperative complication profile. Thus, it is of paramount importance to quantify the added risk of SARS-CoV-2 detection in asymptomatic elective surgical patients.

Interestingly, SARS-CoV-2 detection was associated with a younger age while prolonged length of stay was independently predicted by older age. The authors cannot exclude the possibility that age is a confounding variable. Perhaps SARS-CoV-2 detection carries a risk of prolonged length of stay, but this association was masked by the relative protective effect of younger age in the present cohort. Investigation of a larger cohort would allow for better assessment of confounding or effect-modification by facilitating stratified analysis by age group, or matched analysis comparing SARS-CoV-2 positive patients to age-matched controls. However, the present study design would not allow for such analyses, given that only 30 patients had positive viral detection. Thus, viral detection would be exceedingly rare if stratified by age group and matching 1:2 by age would yield only a 90-patient sample (30 with SARS-CoV-2 detection vs. 60 controls) at most.

Additionally, the risk factors associated with prolonged length of stay (age, preoperative hemoglobin, ASA class 3 or 4, case duration, and estimated blood loss) and with respiratory complications (diabetes and estimated blood loss) are novel findings. Although some risk factors are intuitive, such as age and ASA class 3 and 4, other risk factors, such as preoperative hemoglobin levels, are less obvious for the increased hospital stay. Identification of these risk factors for prolonged hospital stay and respiratory complications will help manage resource for those patients with risk factors.

The factors of male sex, fever, chronic kidney or liver disease, and creatinine concentration have previously been described to be potential risk factors for prolonged (greater than median) length of stay in hospitalized patients with COVID-19 in China (18). However, these risk factors are not specific to elective surgical patients. Risk factors for prolonged length of stay in the elective surgical population include age, functional status, ASA class, need for transfusion, operative time, return to the operating room, and postoperative complications (19). Of note, these risk factors are not unlike the ones identified by the present study. The ARISCAT score for postoperative pulmonary complications derives its calculated risk from the following characteristics: age, preoperative oxygen saturation, preceding respiratory infection, preoperative anemia, surgical incision, duration of surgery, and emergency status (20). Our study suggests that diabetes and estimated blood loss may need to be added for this calculation.

Recent studies have begun to describe the effects of perioperative SARS-CoV-2 infection on hospital and ICU length of stay, surgical complications, and perioperative mortality, however the literature has generally described patients with an established preoperative diagnosis prior to either elective or urgent/emergent surgery.

One multicenter cohort study of hip fracture patients in the UK found that SARS-CoV-2 positive patients had an increased length of postoperative hospital stay compared to controls (13.8 days vs. 6.7 days, *P* < 0.001) (21). These results are somewhat discordant with those of Jungwirth-Weinberger et al. (22), who reported that SARS-CoV-2 was not predictive of post-arthroplasty median length of stay after controlling for confounding variables such as age, sex, and comorbidity index (22). In a cardiac surgery patient cohort, the average length of postoperative stay for SARS-CoV-2 positive patients was 3 days, compared to 1.8 days in controls (*P* = 0.002) (23).

The frequency of ICU admission may also be greater in SARS-CoV-2 positive patients. Among urgent and emergent surgical procedures, the rate of ICU admissions was 36.1% in SARS-CoV-2 positive patients compared to 16.4% in controls (24). A similar difference was identified in non-surgical parturients, who experienced ICU admission rates of 39.5% in SARS-CoV-2 positive patients vs. 17.0% in controls (*P* < 0.01) (25).

In a multicenter sample of 5,479 patients, patients undergoing major elective surgery 0–4 weeks after SARS-CoV-2 infection experienced a significantly greater incidence of postoperative complications such as pneumonia, respiratory failure, and sepsis, while patients 8 + weeks post-SARS-CoV-2 infection did not (9). Across various studies, postoperative SARS-CoV-2 positive patients had more pulmonary and thromboembolic complications (*P* < 0.01) (26), as well as higher risk of cardiac arrest, sepsis/shock, respiratory failure, pneumonia, acute respiratory distress syndrome, and acute kidney injury (24), and higher rates of 30-day pulmonary, septic, and ischemic complications (27). SARS-CoV-2 positive patients undergoing hip surgery had an overall increased risk of postoperative complications (89.0% vs. 35.0%; *P* < 0.001) compared to control patients (21), while elective and emergency surgical patients with perioperative and recent SARS-CoV-2 infection had a higher risk of venous thromboembolism that was independently associated with 30-day mortality (8). Among pediatric patients receiving general anesthesia, SARS-CoV-2 carried a 14.37 odds of pulmonary complications (*P* = 0.02) (28).

Egol et al. (29) reported hip fracture mortality rates of 35.3% in SARS-CoV-2 positive patients compared to 7.1% in SARS-CoV-2 suspected patients and 0.9% in SARS-CoV-2 negative patients (29). Yates et al. (30) reported 44% increased mortality among perioperative cardiac surgery SARS-CoV-2 patients (30), which is nearly as significant as the 4.1, 3.9, and 3.6 odds of mortality described in surgical patients 0–2, 3–4, and 5–6 weeks after SARS-CoV-2 infection (10). One study across 7,402 patients and 50 countries even revealed that 55% of excess postoperative deaths during the pandemic were estimated to be attributable to SARS-CoV-2 (31). Overall mortality was higher among an asymptomatic group of SARS-CoV-2 cardiac surgery patients compared to a propensity-matched group of patients who underwent surgery in the pre-COVID era (23).

This study is limited by a low case number (*n* = 30) of SARS-CoV-2 intraoperative viral detection, and by sampling just two surgical subspecialties at a single medical center. Despite adhering to the sample size guided by the *a priori* power analysis, the present study was likely underpowered to detect a difference between those who did and did not have intraoperative detection of SARS-CoV-2. The prevalence of intraoperative detection was unknown at the time of this study, however, the authors did subjectively believe it would be much higher than 30/1,130 (2.7%) in elective spine and gynecologic surgical patients based on anecdotal observations. The low prevalence of SARS-CoV-2 viral detection precluded the authors from controlling for age with stratified analysis or matched cohorts analysis, as previously discussed. Additionally, the generalizability of these results is limited to asymptomatic patients presenting from home for elective surgeries with planned inpatient admission. As such, the present results cannot be extended to draw conclusions about ambulatory surgery patients, symptomatic patients, or those with a documented preoperative COVID-19 diagnosis. Future studies are needed to appropriately risk stratify and to understand the optimal timing of surgery following asymptomatic SARS-CoV-2 infection. Definitive conclusions cannot be made without large cohorts of SARS-CoV-2 positive patients.

In conclusion, incidental detection of SARS-CoV-2 on intraoperative viral testing does not appear to carry an increased risk for prolonged length of stay, respiratory complications, mortality, or other major adverse events for asymptomatic elective surgical patients. Routine intraoperative testing may help to guide measures aimed at limiting viral transmission, however, the adverse event rate experienced by patients with SARS-CoV-2 detection is no worse than that of controls.

## Data availability statement

The datasets presented in this article are not readily available because data cannot be made publicly available for legal or ethical reasons. Requests to access the datasets should be directed to corresponding author.

## Ethics statement

The studies involving human participants were reviewed and approved by the UVA Institutional Review Board for Health Sciences Research (IRB-HSR# 23998). Written informed consent for participation was not required for this study in accordance with the national legislation and the institutional requirements.

## Author contributions

PC: investigation and writing—original draft. ZK: methodology, software, formal analysis, and writing—original draft. ZZ: conceptualization, resources, writing—review and editing, supervision, and project administration. All authors contributed to the article and approved the submitted version.

## References

[1] Schmidt A Módolo N de Amorim C Simões C Kraychette D Joaquim E Two years of the COVID-19 pandemic: an anesthesiology perspective. *Braz J Anesthesiol.* (2022) 72:165–8. 10.1016/j.bjane.2022.02.004 35189166 PMC8856750

[2] COVIDSurg Collaborative. Elective surgery cancellations due to the COVID-19 pandemic: global predictive modelling to inform surgical recovery plans. *Br J Surg.* (2020) 107: 1440–9. 32395848 10.1002/bjs.11746 PMC7272903

[3] COVIDSurg Collaborative. Projecting COVID-19 disruption to elective surgery. *Lancet.* (2022) 399:233–4. 34922640 10.1016/S0140-6736(21)02836-1 PMC8676422

[4] COVIDSurg Collaborative. Mortality and pulmonary complications in patients undergoing surgery with perioperative SARS-CoV-2 infection: an international cohort study. *Lancet.* (2020) 396: 27–38. 32479829 10.1016/S0140-6736(20)31182-X PMC7259900

[5] Lei S Jiang F Su W Chen C Chen J Mei W Clinical characteristics and outcomes of patients undergoing surgeries during the incubation period of COVID-19 infection. *EClinicalMedicine.* (2020) 21:100331. 10.1016/j.eclinm.2020.100331 32292899 PMC7128617

[6] Wang D Hu B Hu C Zhu F Liu X Zhang J Clinical characteristics of 138 hospitalized patients with 2019 novel coronavirus-infected pneumonia in Wuhan. China. *JAMA.* (2020) 323:1061–9. 10.1001/jama.2020.1585 32031570 PMC7042881

[7] Huang C Wang Y Li X Ren L Zhao J Hu Y Clinical features of patients infected with 2019 novel coronavirus in Wuhan. China. *Lancet.* (2020) 395:497–506. 10.1016/S0140-6736(20)30183-5 31986264 PMC7159299

[8] COVIDSurg Collaborative, GlobalSurg Collaborative. SARS-CoV-2 infection and venous thromboembolism after surgery: an international prospective cohort study. *Anaesthesia.* (2022) 77:28–39. 34428858 10.1111/anae.15563 PMC8652887

[9] Deng J Chan J Potter A Chen Y Sandhu H Panda N The risk of postoperative complications after major elective surgery in active or resolved COVID-19 in the United States. *Ann Surg.* (2022) 275:242–6. 10.1097/SLA.0000000000005308 34793348 PMC8745943

[10] COVIDSurg Collaborative, GlobalSurg Collaborative. Timing of surgery following SARS-CoV-2 infection: an international prospective cohort study. *Anaesthesia.* (2021) 76:748–58. 33690889 10.1111/anae.15458 PMC8206995

[11] Sayed I Bhalala U Strom L Tripathi S Kim J Michaud K Gastrointestinal manifestations in hospitalized children with acute SARS-CoV-2 infection and multisystem inflammatory condition: an analysis of the VIRUS COVID-19 registry. *Pediatr Infect Dis J.* (2022). [Epub ahead of print]. 10.1097/INF.0000000000003589 35622434 PMC9359679

[12] Chameply S *pwr: basic functions for power analysis. R package version 1.3-0.* (2020). Available online at: https://CRAN.R-project.org/package=pwr (accessed June 18, 2022).

[13] R Core Team. *R: a language and environment for statistical computing.* Vienna: R Foundation for Statistical Computing (2022).

[14] Therneau T Grambsch P. *Modeling survival data: extending the cox model.* New York, NY: Springer (2000). 10.1007/978-1-4757-3294-8

[15] Therneau T. *A package for survival analysis in R. R package version 3.2-7.* (2020). Available online at: https://CRAN.R-project.org/package=survival (accessed July 15, 2022).

[16] Harrell F Jr. *rms: regression modeling strategies. R package version 6.0-0.* (2020). Available online at: https://CRAN.R-project.org/package=rms (accessed June 19, 2022).

[17] Lieberman N Racine A Nair S Semczuk P Azimaraghi O Freda J Should asymptomatic patients testing positive for SARS-CoV-2 wait for elective surgical procedures? *Br J Anaesth.* (2022) 128:e311–4. 10.1016/j.bja.2022.02.005 35277245 PMC8847097

[18] Guo A Lu J Tan H Kuang Z Luo Y Yang T Risk factors on admission associated with hospital length of stay in patients with COVID-19: a retrospective cohort study. *Sci Rep.* (2021) 11:7310. 10.1038/s41598-021-86853-4 33790365 PMC8012638

[19] Collins T Daley J Henderson W Khuri S. Risk factors for prolonged length of stay after major elective surgery. *Ann Surg.* (1999) 230:251. 10.1097/00000658-199908000-00016 10450740 PMC1420868

[20] Canet J Gallart L Gomar C Paluzie G Vallès J Castillo J Prediction of postoperative pulmonary complications in a population-based surgical cohort. *Anesthesiology.* (2010) 113:1338–50. 10.1097/ALN.0b013e3181fc6e0a 21045639

[21] Kayani B Onochie E Patil V Begum F Cuthbert R Ferguson D The effects of COVID-19 on perioperative morbidity and mortality in patients with hip fractures. *Bone Joint J.* (2020) 102-B:1136–45. 10.1302/0301-620X.102B9.BJJ-2020-1127.R1 32634023

[22] Jungwirth-Weinberger A Boettner F Kapadia M Diane A Chiu Y Lyman S History of COVID-19 was not associated with length of stay or in-hospital complications after elective lower extremity joint replacement. *Arthroplast Today.* (2022) 13:109–15. 10.1016/j.artd.2021.11.021 34909457 PMC8660178

[23] Barkhordari K Khajavi M Bagheri J Nikkhah S Shirzad M Barkhordari S Early respiratory outcomes following cardiac surgery in patients with COVID-19. *J Card Surg.* (2020) 35:2479–85. 10.1111/jocs.14915 32789988 PMC7436810

[24] Knisely A Zhou Z Wu J Huang Y Holcomb K Melamed A Perioperative morbidity and mortality of patients with COVID-19 who undergo urgent and emergent surgical procedures. *Ann Surg.* (2021) 273:34–40. 10.1097/SLA.0000000000004420 33074900 PMC7737869

[25] DeBolt C Bianco A Limaye M Silverstein J Penfield C Roman A Pregnant women with severe or critical coronavirus disease 2019 have increased composite morbidity compared with nonpregnant matched controls. *Am J Obstet Gynecol.* (2021) 224:510.e1–12. 10.1016/j.ajog.2020.11.022 33221292 PMC7677036

[26] Jonker P van der Plas W Steinkamp P Poelstra R Emous M van der Meij W Perioperative SARS-CoV-2 infections increase mortality, pulmonary complications, and thromboembolic events: a dutch, multicenter, matched-cohort clinical study. *Surgery.* (2021) 169:264–74. 10.1016/j.surg.2020.09.022 33158548 PMC7513767

[27] Lal B Prasad N Englum B Turner D Siddiqui T Carlin M Periprocedural complications in patients with SARS-CoV-2 infection compared to those without infection: a nationwide propensity-matched analysis. *Am J Surg.* (2021) 222:431–7. 10.1016/j.amjsurg.2020.12.024 33384154 PMC7836786

[28] Saynhalath R Alex G Efune P Szmuk P Zhu H Sanford E. Anesthetic complications associated with severe acute respiratory syndrome coronavirus 2 in pediatric patients. *Anesth Analg.* (2021) 133:483–90. 10.1213/ANE.0000000000005606 33886516

[29] Egol K Konda S Bird M Dedhia N Landes E Ranson R Increased mortality and major complications in hip fracture care during the COVID-19 pandemic: a New York city perspective. *J Orthop Trauma.* (2020) 34:395–402. 10.1097/BOT.0000000000001845 32482976 PMC7302075

[30] Yates M Balmforth D Lopez-Marco A Uppal R Oo A. Outcomes of patients diagnosed with COVID-19 in the early postoperative period following cardiac surgery. *Interact Cardiovasc Thorac Surg.* (2020) 31:483–5. 10.1093/icvts/ivaa143 32791519 PMC7454553

[31] STARSurg Collaborative and COVIDSurg Collaborative. Death following pulmonary complications of surgery before and during the SARS-CoV-2 pandemic. *Br J Surg.* (2021) 108:1448–64. 34871379 10.1093/bjs/znab336 PMC10364875

