# Supplementary material for: Positive SARS-CoV-2 detection on intraoperative nasopharyngeal viral testing is not associated with worse outcomes for asymptomatic elective surgical patients
Source: Front Med (Lausanne). 2022 Dec 21;9:1065625. doi: 10.3389/fmed.2022.1065625 (PMC9810621; doi:10.3389/fmed.2022.1065625)
Supplement: Supplementary file 1 [file Table_1.DOCX]

Supplementary Material

# Supplementary Table 1. International Classification of Diseases 10 (ICD-10) indices.

| Characteristic | ICD-10 Code | Diagnosis Name |
| --- | --- | --- |
| COPD | J44.0 | Chr Obstructive Pulmon Disease With (Acute) Lower Resp Infct |
|  | J44.1 | Chronic Obstructive Pulmonary Disease W (Acute) Exacerbation |
|  | J44.9 | Chronic Obstructive Pulmonary Disease, Unspecified |
| Diabetes mellitus | E10.42 | Type 1 Diabetes Mellitus With Diabetic Polyneuropathy |
|  | E10.43 | Type 1 Diabetes W Diabetic Autonomic (Poly)Neuropathy |
|  | E10.65 | Type 1 Diabetes Mellitus With Hyperglycemia |
|  | E10.9 | Type 1 Diabetes Mellitus Without Complications |
|  | E11.21 | Type 2 Diabetes Mellitus With Diabetic Nephropathy |
|  | E11.22 | Type 2 Diabetes Mellitus W Diabetic Chronic Kidney Disease |
|  | E11.36 | Type 2 Diabetes Mellitus With Diabetic Cataract |
|  | E11.39 | Type 2 Diabetes W Oth Diabetic Ophthalmic Complication |
|  | E11.40 | Type 2 Diabetes Mellitus With Diabetic Neuropathy |
|  | E11.41 | Type 2 Diabetes Mellitus With Diabetic Mononeuropathy |
|  | E11.42 | Type 2 Diabetes Mellitus With Diabetic Polyneuropathy |
|  | E11.43 | Type 2 Diabetes W Diabetic Autonomic (Poly)Neuropathy |
|  | E11.49 | Type 2 Diabetes W Oth Diabetic Neurological Complication |
|  | E11.51 | Type 2 Diabetes W Diabetic Peripheral Angiopath W/O Gangrene |
|  | E11.65 | Type 2 Diabetes Mellitus With Hyperglycemia |
|  | E11.69 | Type 2 Diabetes Mellitus With Other Specified Complication |
|  | E11.9 | Type 2 Diabetes Mellitus Without Complications |
| Hypertension | I10 | Essential (Primary) Hypertension |
| Respiratory complication | J95.811 | Postprocedural Pneumothorax |
|  | J95.851 | Ventilator Associated Pneumonia |
|  | J96.00 | Acute Respiratory Failure, Unsp W Hypoxia Or Hypercapnia |
|  | J96.01 | Acute Respiratory Failure With Hypoxia |
|  | J98.11 | Atelectasis |
|  | J96.02 | Acute Respiratory Failure With Hypercapnia |
| Pulmonary embolism | I26.09 | Other Pulmonary Embolism With Acute Cor Pulmonale |
|  | I26.92 | Saddle Embolus Of Pulmonary Artery W/O Acute Cor Pulmonale |
|  | I26.93 | Single Subsegmental Pulmonary Embolism Without Acute Cor Pulmonale |
|  | I26.94 | Multiple Subsegmental Pulmonary Emboli Without Acute Cor Pulmonale |
|  | I26.99 | Other Pulmonary Embolism Without Acute Cor Pulmonale |
| Sepsis | A41.52 | Sepsis Due To Pseudomonas |
|  | A41.53 | Sepsis Due To Serratia |
|  | A41.9 | Sepsis, Unspecified Organism |
|  | R65.21 | Severe Sepsis With Septic Shock |
| Myocardial infarction | I21.A1 | Myocardial Infarction Type 2 |
| Stroke | I63.89 | Other Cerebral Infarction |
|  | I63.9 | Cerebral Infarction, Unspecified |
